# Supplementary material for: Oscillations in networks of networks stem from adaptive nodes with memory
Source: Sci Rep. 2017 Jun 2;7:2700. doi: 10.1038/s41598-017-02814-w (PMC5457433; doi:10.1038/s41598-017-02814-w)
Supplement: Supplementary file 1 — Supplementary Material [file 41598_2017_2814_MOESM1_ESM.pdf]

# Supplementary: Oscillations in networks of networks stem from adaptive nodes with memory

**Amir Goldental†, Herut Uzan†, Shira Sardi and Ido Kanter***

†These authors contributed equally to this work.

*Correspondence: ido.kanter@biu.ac.il

## Normalization of $h_k(i,m)$

The $h_k(i,m)$ that appears in Eq. (8) is normalized,

$$h_k(i,m) = h'_k(i,m) / \left[ q_k(i) + \sum_{m=1}^{(f_c d)^{-1}} h'_k(i,m) \right] \qquad \text{(S1)}$$

where $h'_k(i,m)$ is the $h_k(i,m)$ presented in Eq. (6) and

$$q_k(i) = 1 - \sum_{m=1}^{(f_c d)^{-1}} \langle h'_k(i,m) \rangle + \xi_k^q(i) \qquad \text{(S2)}$$

where the last term stands for random fluctuations in finite networks which scale with $N^{-0.5}$ (Gaussian random variable with a variance of $<q_k(i)>\cdot(1-<q_k(i)>)/(p^{st}{}_k(i)\cdot C_k \cdot N)$ and a zero mean).

## α>0

In case $\alpha>0$ the response probability is given by Eq. (3), which can be rewritten as

$$p_\alpha^{sp}(\Delta t_2, \dots, \Delta t_n) = \alpha \cdot p_\alpha^{sp}(\Delta t_2, \dots, \Delta t_{n-1}) + (1-\alpha) \cdot p^{sp}(\Delta t_n) \qquad \text{(S3)}$$

The first term in the right hand side can be approximated, by averaging, as

$$\alpha \cdot p_\alpha^{sp}(\Delta t_2, \dots, \Delta t_{n-1}) \approx \alpha \cdot p_\alpha^{sp}\left(\frac{d}{k\langle \mathrm{R} \rangle_i}, \dots, \frac{d}{k\langle \mathrm{R} \rangle_i}\right) = \alpha p^{sp}\left(\frac{d}{k\langle \mathrm{R} \rangle_i}\right) \qquad \text{(S4)}$$

where $<R>_i$ is given by solving Eqs. (4)-(8) while setting $R(i)=<R>_i$ for all $i$ and neglecting all the noise terms.

## Network of networks

Here each sub-network, $g$, has a connectivity probability mass function $C_{g,k1\dots kn}$ which gives the probability for a neuron from sub-network $g$ to receive stimulations from $k_1,\dots,k_n$ neurons from sub network $1,\dots,n$, respectively. For example, the $C_{g,k1\dots kn}$ which describes the system in Fig. 4 in the manuscript is given by

$$C_{g,k_1,\dots,k_n} = \delta_{g,1}\delta_{k_1,1}\delta_{k_2,0}\delta_{k_3,0}\delta_{k_4,2} + \delta_{g,2}\delta_{k_1,2}\delta_{k_2,1}\delta_{k_3,0}\delta_{k_4,0}$$
$$+\delta_{g,3}\delta_{k_1,0}\delta_{k_2,2}\delta_{k_3,1}\delta_{k_4,2} + \delta_{g,4}\delta_{k_1,0}\delta_{k_2,0}\delta_{k_3,2}\delta_{k_4,1} \qquad \text{(S5.1)}$$

Additionally, the delays between network $g$ and $g'$ are now given by the matrix $d_{g,g'}$, e.g. in Fig. 4

$$d_{g,g'} = [10\,ms]\delta_{g,g'} + [20\,ms](1-\delta_{g,g'}) \quad \text{(S5.2)}$$

All nodes that share the same connectivity properties ($g,k_1,\ldots,k_n$) have the same stimulation probability $p^{st}_{g,k1,\ldots,kg}$ and susceptibility $\chi_{g,k1,\ldots,kg}$. As a result, the $R$ of group $g$, denoted as $R_g$, is given by:

$$R_g(i) = \sum_{k_1=0}^{k_{max}} \ldots \sum_{k_n=0}^{k_{max}} C_{g,k_1,\ldots,k_n} \cdot p^{st}_{g,k_1,\ldots,k_n}(i) \cdot \chi_{g,k_1,\ldots,k_n}(i) \qquad \text{(S5.3)}$$

where

$$p^{st}_{g,k_1,\ldots,k_n}(i) = 1 - \prod_{g'=1}^{n}\left[1 - R_g\left(i - d_{g,g'}\right)\right]^{k_g} \cdot (1 - f_{ext} \cdot d) + \xi^{st}_{k_1,\ldots,k_n}(i) \qquad \text{(S5.4)}$$

and the susceptibility is given by

$$\chi_{g,k_1,\ldots,k_n}(i) = 1 - \sum_{m=1}^{(f_c d)^{-1}} (1 - m \cdot d \cdot f_c) h_{g,k_1,\ldots,k_n}(i,m) \quad \text{(S5.5)}$$

where

$$h_{g,k_1,\ldots,k_n}(i,m) = p^{st}_{g,k_1,\ldots,k_n}(i-m) \prod_{n=1}^{m-1}\left[1 - p^{st}_{g,k_1,\ldots,k_n}(i-n)\right] + \xi^{h}_{g,k_1,\ldots,k_n}(i,m) \quad \text{(S5.6)}$$

Similar to the manuscript, $h_{g,k1,\ldots,kn}$ is normalized, the variance of the stochastic term in Eq. S5.4 is $\langle p^{st}_{g,k1,\ldots,kg}(i)\rangle \cdot (1-\langle p^{st}_{g,\, k1,\ldots,kg}(i)\rangle)/(C_{g,k1,\ldots,kg} \cdot N)$ and the variance of the stochastic term in Eq. S5.6 is $\langle h_{g,k1,\ldots,kg}(i,m)\rangle \cdot (1-\langle h_{g,k1,\ldots,kg}(i,m)\rangle)/(p^{st}_{g,k1,\ldots,kg}(i) \cdot C_{g,k1,\ldots,kg} \cdot N)$
